# Key lessons from Liberia for successful partnerships toward universal health coverage in low-resource settings

**DOI:** 10.1093/inthealth/ihae028

**Published:** 2024-04-12

**Authors:** Tiawanlyn G Godwin-Akpan, Rosalind McCollum, Jerry Kollie, Hannah Berrian, Wede Seekey-Tate, John S Smith, Fasseneh Zeela Zaizay, Shahreen Chowdhury, Karsor K Kollie, Emerson J Rogers, Colleen B M C Parker, Georgina V K Zawolo, Anna Wickenden, Laura Dean, Sally Theobald

**Affiliations:** American Leprosy Missions, Accra, Ghana; Liverpool School of Tropical Medicine, Liverpool, UK; University of Liberia Pacific Institute for Research and Evaluation – UL-PIRE, University of Liberia, Monrovia, Liberia; University of Liberia Pacific Institute for Research and Evaluation – UL-PIRE, University of Liberia, Monrovia, Liberia; University of Liberia Pacific Institute for Research and Evaluation – UL-PIRE, University of Liberia, Monrovia, Liberia; University of Liberia Pacific Institute for Research and Evaluation – UL-PIRE, University of Liberia, Monrovia, Liberia; Actions Transforming lives, Oldest Congo Town, Monrovia, Liberia; Liverpool School of Tropical Medicine, Liverpool, UK; Neglected Tropical Diseases Program, Ministry of Health, Oldest Congo Town, Monrovia, Liberia; Neglected Tropical Diseases Program, Ministry of Health, Oldest Congo Town, Monrovia, Liberia; Research Unit, Ministry of Health, Oldest Congo Town, Monrovia, Liberia; University of Liberia Pacific Institute for Research and Evaluation – UL-PIRE, University of Liberia, Monrovia, Liberia; WaterAid Liberia, Sinkor, Monrovia, Liberia; Liverpool School of Tropical Medicine, Liverpool, UK; Effect Hope, Markham, Ontario, Canada; Liverpool School of Tropical Medicine, Liverpool, UK; Liverpool School of Tropical Medicine, Liverpool, UK

**Keywords:** access, national governments, health system, Liberia, neglected tropical diseases, persons affected, universal health coverage

## Introduction

Global health actors and stakeholders advocate for universal health coverage (UHC) for all, as emphasized within the United Nation's Sustainable Development Goals (SDGs; goal 3).^1^ The attainment of UHC is shaped by access, quality and affordability of health services; health systems and the six health system building blocks of the World Health Organization (WHO) are critical here.^2,3^ However, strengthening these building blocks in isolation is insufficient to achieve UHC in resource-challenged settings due to the complex interplay of social and political factors that influence healthcare expansion and access. Thus, achieving UHC relies on collaboration and relationships within and across all health system levels, actors, sectors, diseases, institutions and community structures. However, minimal evidence exists about identifying, building and strengthening these relationships and connections to build an effective, efficient and resilient health system for neglected tropical diseases (NTDs).

NTDs are a group of 20 diseases that lead to visible impairments, disabilities and psychosocial impacts, contributing to a cycle of ill health and poverty and reducing quality of life.^4^ Control and management of NTDs have been described as a critical litmus test for attaining universal health coverage as they impact the poorest, vulnerable and hard-to-reach communities.^2^ However, stigma, low knowledge of NTD diagnoses and management among health workers and communities and vertical disease-specific programs outside the routine health system hinder achieving UHC for persons affected by NTDs. The WHO, in its new 2030 road map, has set ambitious targets, including mainstreaming NTD services within the health system to control and eliminate NTDs.^4^

**Figure 1.**
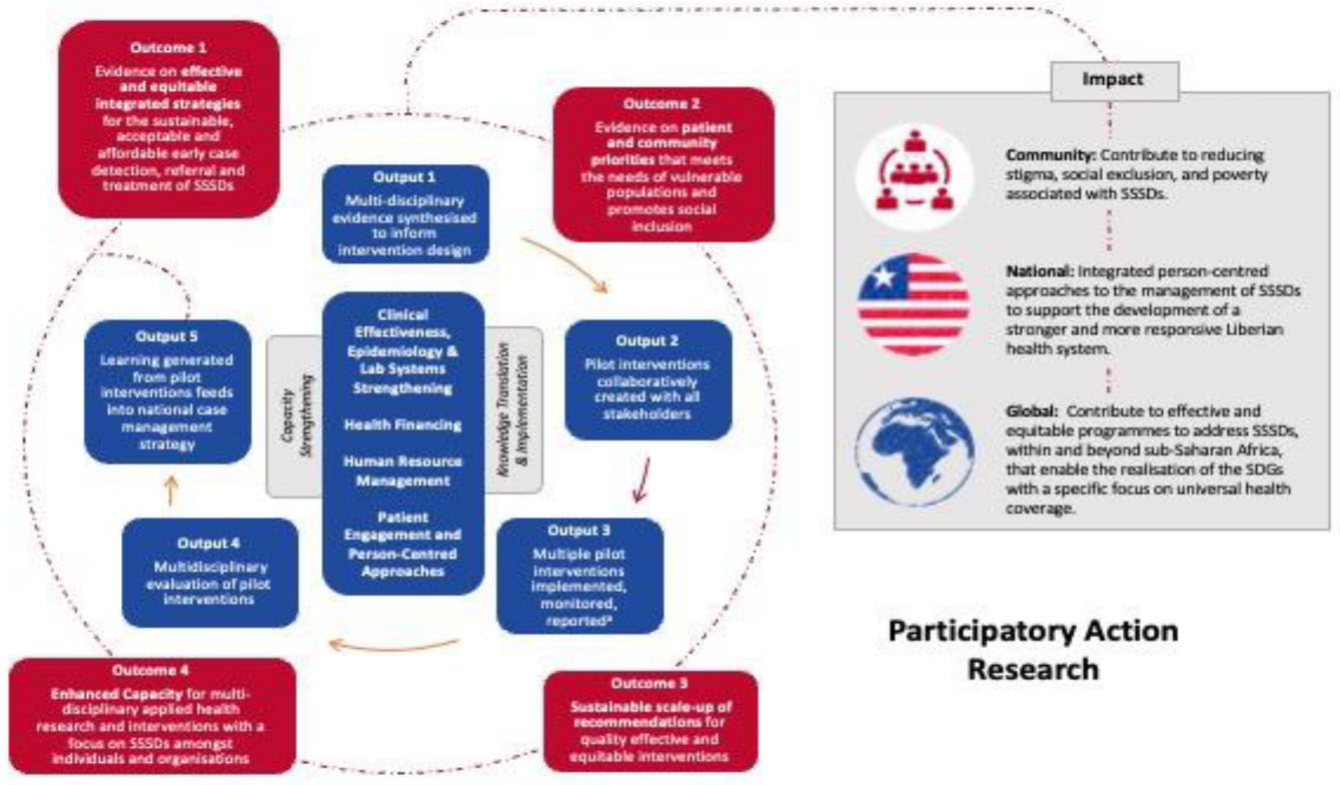
REDRESS theory of change summary Phase 1: Formative phase • During this phase, we evaluated existing integrated approaches for the early detection, referral and treatment of SSSDs from the perspective of our core themes. Phase 2: Planning • During this phase, we worked with health systems actors and people affected by stigmatising skin diseases to co-create new and adapt existing interventions. Phase 3: Action • During this phase, we implemented co-created interventions through existing health systems and community infrastructure. Phase 4: Observation and reflection • During this phase, we conducted a multi-disciplinary evaluation of intervention bundles aligned to our core themes to make recommendations for quality improvement and scale-up of integrated SSSD interventions in Liberia. Phase 5: Knowledge translation and policy change This phase was implemented throughout the lifetime of REDRESS along with the MoH to scale up and embed successful interventions within routine health systems management of SSSDs and to make recommendations for the integrated management of SSSDs across the West African region.

In 2016, Liberia became one of the first countries to develop an integrated strategy for case management of NTDs (CM-NTDs). This involved embedding existing NTD structures within the health system, including integrating training for case detection and referral of targeted NTDs into the national community health training manual, production of an integrated skin NTDs training manual for primary healthcare workers and the integration of CM-NTD data into the national health information system (HIS) through the district health information system 2 (DHIS2) platform. Liberia piloted this 5-y strategy in five counties. After 3 y of implementing an integrated approach to case management of NTDs, the national NTDs program, in collaboration with its partners, developed the Reducing the Burden of Severe Stigmatising Skin Diseases (REDRESS) research program to evaluate existing approaches and make recommendations for quality improvement and scale-up to ensure equitable service access, thus contributing toward attaining UHC for persons affected by NTDs in Liberia. Figure 1 presents the REDRESS theory of change.

REDRESS expanded on a collaborative approach to strengthening the health system by introducing new partnerships, including those with mental health actors, community members, persons affected by NTDs and traditional and faith healers, to understand how these relationships impact UHC within counties that had previously piloted integrated case management (quality improvement) and those with no previous pilot (scale-up). Participatory action research methodologies guide this implementation research study, which deploys a holistic approach to person-centred care for persons affected by NTDs through intrasectoral collaboration between NTDs, community health and mental health and other departments within the Ministry of Health in Liberia and persons affected by NTDs. Severe stigmatising skin diseases (SSSDs) in the REDRESS study are synonymous with case management NTDs, which include leprosy, onchocerciasis, Buruli ulcer, yaws and clinical presentations of lymphatic filariasis, specifically lymphoedema and hydrocele.

This article distils practical lessons and frontline experiences from ongoing implementation research, discusses the potential partnerships toward achieving UHC and provides recommendations for health system strengthening to guide policymakers and national governments.

### Strengthening the health system through collaboration: four key lessons

#### Participation of persons affected

The REDRESS project is designed as a participatory action research study that embeds persons affected by skin NTDs throughout the study design stages. During the exploratory and intervention development phases, participants included persons affected who provided personal experiences on their needs, challenges and capabilities in accessing healthcare. This led to the formation of peer support groups as part of the intervention in each research county. These groups provide a space for persons affected to meet and share experiences. REDRESS empowered health workers at primary health facilities in the three research counties to train 20 persons affected by NTDs per county for case identification and referral, psychosocial support, complementary peer support to each other and advice and guidance on self-care, income generation and community awareness. As health facilities diagnosed new NTD patients, they were referred to their local support groups. The peer support groups complemented the work of the trained community health workers by providing information on suspected cases that may otherwise not have been found due to fear of stigma or misconception about managing these conditions. We found that peer support groups help dispel the misunderstanding and stigma around NTDs and promote positive health-seeking behaviours.

#### Integrating NTDs, mental health and community health

People affected by NTDs often experience mental health conditions, which may affect their willingness to seek care and adhere to treatment regimens; however, this multimorbidity is frequently missed, as many health workers are not trained to consider or identify mental health conditions.^5^ Leveraging on the existing relationship between NTDs and community health programs, mental health, as a cross-cutting issue, was integrated into the NTD training package, diagnostic pathway, data management and reporting. Community health workers were trained to provide basic psychological support and identify signs of stress, isolation and other mental health issues during their door-to-door active case searching for suspected NTDs within their communities. Furthermore, health workers were also trained (based on the WHO Mental Health Gap Action Programme training) on how to screen for depression and anxiety using the 9-item Patient Health Questionnaire and 7-item generalised anxiety disorder scale, which were adapted and validated for use in Liberia. Patients suspected of having a mental health condition were referred to mental health clinicians for further support within the existing Liberian mental health referral pathway. This is the first time Liberia has integrated mental health, NTDs and community health. This intersectoral partnership promotes resources and information sharing at all levels, increases care coverage and improves the likelihood of being diagnosed and managed for either or both conditions.

#### The partnership between the formal and informal health sectors

Our initial research showed that traditional and faith healers are often the first point of care for persons affected by NTDs. This is due to multiple misconceptions about the cause of these conditions, which leads to stigmatization exacerbated by mistrust in formal health providers. The project trained 294 traditional healers (spiritual and herbal medicine) and faith healers to identify suspected NTD cases and refer them to the health facility. Once a patient is diagnosed, feedback is provided to the traditional or faith healer who referred the case, and they are also encouraged to provide moral support through visitation. Likewise, if appropriate, persons diagnosed at the health facility are advised to seek moral support from traditional or faith healers. Health workers also work alongside traditional and faith healers to identify persons affected and support them with medicine regimens. The partnership between the formal and informal sectors improves the identification and early referral of cases, promotes awareness and fosters holistic care by providing spiritual, emotional and physical support for individuals impacted by NTDs. This person-centred holistic approach to care aligns with the principles of UHC for all.

#### Multidisciplinary technical advisory boards

REDRESS includes a multidisciplinary technical advisory board at the national Ministry of Health. This group of experts from diverse backgrounds, including mental health, supply chain, policy and planning, community health and health services and medical and paramedical regulatory boards, guides the proposed interventions and shares information across the health system. Several smaller technical working groups were also established to improve aspects of intervention development, e.g. a human resource management group. The Community Advisory Board consists of 16 leaders from various sectors, including persons affected by skin NTDs, traditional and religious organizations, gender and youth-based, civil society organizations, livelihood support, business etc., to provide holistic guidance for the interventions within each county. These advisory groups have improved the overall governance of the REDRESS project, provided insights into best practices, held implementers accountable and supported sustainability. Since NTD interventions are cross-cutting with other sectors and diseases, such as education, disability inclusion and mental health, a multidisciplinary governance structure ensures the visibility of NTDs and prioritizes national and subnational policies and plans toward attaining UHC.

## Conclusions

The multifaceted collaboration implemented through the REDRESS project reduces barriers to accessing care and progresses toward UHC. It aligns with the strategic pillars of the WHO NTD roadmap 2021–2030,^6^ specifically pillar 2: intensifying cross-cutting approaches to prevent, control and eliminate NTDs. Leaving no one behind means all disease conditions must be prioritized and policies and plans must be inclusive and reflect collaboration across multiple stakeholders to improve access to essential services for all. These four lessons for successful partnership in health programming can be applied to all disease conditions, as they cut across the social determinants of health. Governments, policymakers and practitioners in low-resource settings should adapt these lessons to their unique socio-political environment as key strategies to create an environment that fosters collaboration and partnerships across programs, sectors and administrative structures to achieve and sustain UHC for all.

## Data Availability

Data analysed for this manuscript are available from the corresponding author on reasonable request.
